# Regulation and governance for the implementation and management of point-of-care testing in Australia: a scoping review

**DOI:** 10.1186/s12889-025-21894-2

**Published:** 2025-02-24

**Authors:** Jacqueline Prestedge, Claire Kaufman, Deborah A. Williamson

**Affiliations:** 1 Department of Infectious Diseases, The University of Melbourne at The Peter Doherty Institute for Infection and Immunity, Melbourne, VIC Australia; 2 Victorian Infectious Diseases Reference Laboratory, The Royal Melbourne Hospital at The Peter Doherty Institute for Infection and Immunity, Melbourne, VIC Australia; 3 School of Medicine, University of St Andrews, Fife, Scotland KY16 9TF UK; 4 MRC- University of Glasgow Centre for Virus Research, Glasgow, Scotland G61 1QH UK

**Keywords:** Point of care testing, Regulation, Governance, Quality, Pathology

## Abstract

**Background:**

Point-of-care testing (PoCT) is an increasingly important diagnostic tool in the healthcare system for accessible pathology testing in hospital, primary care, and community care settings. Clear regulation and governance models are important to ensure quality of PoCT results for patient care.

**Methods:**

This review aimed to identify existing regulation and guidelines for management of PoCT and how this has been implemented within Australian healthcare services. We conducted a search of academic publications in PubMed and grey literature (national, state, and independent organisations) and other publicly available information from internet searches for governance of PoCT in Australia. Relevant data from these sources were extracted and narratively synthesised.

**Results:**

Forty-seven sources (17 studies from PubMed, 30 grey literature) were included in the final review. Of the grey literature sources, fifteen current PoCT governance documents comprising of six standards, five guidelines and four frameworks at the international, national and jurisdictional level were included with an increasing number of grey literature sources since the onset of the COVID-19 pandemic in 2020. The seventeen included research articles were categorised according to implementation barriers and facilitators with the themes of workforce, clinical governance, PoCT workflow, and cost. An understanding of the clinical and cultural context for PoCT was the most frequently reported facilitator of PoCT, while the most frequently reported barrier was related to inadequate data management.

**Conclusion:**

This review demonstrated limited and inconsistent sources on regulatory and governance models for implementing and managing PoCT in Australia. Identified PoCT programs showcased diverse implementation and governance models to support quality PoCT, with few reporting formal accreditation. Streamlined, practical regulation and governance for PoCT may increase adoption across healthcare settings while ensuring quality results and meeting the needs of patients and healthcare practitioners.

**Supplementary Information:**

The online version contains supplementary material available at 10.1186/s12889-025-21894-2.

## Background

Medical pathology is an integral component of healthcare, providing critical information to support medical decision making and patient care. The traditional model for delivery of medical pathology has been centred around testing performed in a medical laboratory setting by pathology services with prerequisite expertise, equipment, training, quality systems and accreditation to ensure high-quality results [1]. Prompt and reliable diagnosis is crucial to the overall function and cost-effectiveness of healthcare systems [2]. In recent decades, the need for timely access to results has led to the development of portable and fast diagnostics, which can be conducted in close proximity to a patient by a healthcare worker, usually outside of a traditional laboratory, commonly known as point-of-care testing (PoCT) [3].

The integration of PoCT into clinical practice can result in many benefits, making it a valuable addition to patient care [4]. Rapidly generated results can facilitate timely clinical decisions, including providing appropriate treatments without loss to follow up and reducing unnecessary health interventions which burdens the healthcare system. PoCT can improve healthcare access in remote settings and for vulnerable populations where patients can receive diagnosis and possibly treatment in a single medical visit, alleviating anxiety and uncertainty, improving communication, and promoting better engagement with healthcare services [5]. While technological advancements have produced more robust and accurate PoCT devices, their unregulated use may increase the likelihood of diagnostic errors and potentially compromise result quality [6]. Subsequent impacts on critical elements such as interpretation, diagnosis, notification, data management and reporting for clinical and public health responses underscore the need for appropriate regulation and governance [7]. These provide safeguards and ensure effective management for successful PoCT implementation.

During the COVID-19 pandemic, the demand and reliance among clinicians and consumers on PoCT for screening and diagnosis of SARS-CoV-2 increased, and was further fuelled by challenges in laboratory capacity and limited access to healthcare services [8]. Governments and organisations were required to rapidly consider logistical implications, risks, and issues regarding quality, reporting and data use, and to act quickly to guide the use and management of COVID-19 PoCT devices [9, 10]. Amidst the evolving knowledge and evidence surrounding COVID-19 and the use of PoCT during the pandemic, clear governance models were needed to ensure standardised implementation, quality assurance, and optimal patient outcomes from rapid COVID-19 testing. In Australia, pathology services are administered under the Health Insurance Act (1973), which states that laboratories must be accredited under the National Pathology Accreditation Advisory Council’s (NPAAC) standards to be eligible to receive a financial benefit for services under the Medicare Benefits Scheme (MBS). This includes services related to PoCT, meaning laboratories and PoCT providers are required to meet the same minimum standards for governance, evaluation, and management responsibilities [11, 12]. The NPAAC’s ‘Requirements for Point-of-care testing’ provides the national standards against which healthcare services are assessed and accredited by the National Association of Testing Authorities (NATA) in the use of PoCT in Australia [3]. The NPAAC standards were developed with reference to the former International Organization for Standardization (ISO) 15,189:2012 and ISO 22870:2016 standards for medical laboratories which have now been incorporated into ISO 15189:2022. This consolidation may encourage more laboratories globally to extend their existing ISO 15189 accreditation for laboratory-based tests to include PoCT [13, 14]. The NPAAC document adopts a simplified, risk-based approach under a ‘clinical governance’ framework and does not reproduce the ISO standards in full. Regulation of PoCT devices differ globally. For example, the USA Food and Drug Administration categorises devices based on complexity under the Clinical Laboratory Improvement Amendments (CLIA) program [15]. High and moderate complexity tests require accreditation, while simpler ‘CLIA waived’ tests do not. Manufacturers may categorise PoC tests as moderate complexity or waived, with sites conducting waived tests only requiring a certificate of waiver to operate without oversight. Conversely, the Australian accreditation process is designed to cover all PoCT services, regardless of the complexity of PoC tests. In Australia, PoC tests are subject to review of evidence supporting the safety and performance of devices before approval by the Therapeutic Goods Administration (TGA) and subsequent listing on the Australian Register of Therapeutic Goods (ARTG) for supply within Australia [16].

Beyond marketing approval by the TGA, regulation and guidance on what, when, where and how PoC tests are performed i.e. the selection, implementation, quality assurance and reporting of PoCT usage varies across jurisdictions and health sectors. While there are many studies that report PoCT usage, the uptake of widespread PoCT may be hindered in some healthcare settings by the lack of clinical guidance, management challenges, reliable quality control and clear financial reimbursement. To date, published literature on PoCT implementation has largely been focused on evaluations of specific PoC devices or a single disease focus. Few efforts have been made towards understanding the healthcare regulatory environment that PoCT is operating in, with little clarity on how PoCT is being managed in routine clinical care. This review aimed to identify existing regulatory and governance models for the implementation and management of PoCT in Australian healthcare settings.

## Methods

### Purpose of the scoping review

In this scoping review, we focused on the governance of PoCT in Australia, aiming to identify existing policies, gaps, and opportunities in guidance. The primary objective of this review was to explore the various governance models and quality/ regulatory guidance provided for PoCT in healthcare service delivery. Additionally, for studies describing PoCT governance models, a secondary objective was to determine the reported facilitators or barriers influential to PoCT operations. By examining these aspects, we sought to provide insights which can inform future policies supporting the implementation of PoCT in diverse healthcare settings, including hospitals, primary care and general practice (GP), community care, pharmacy, and others. Given the limited information available on the governance of PoCT in Australian healthcare settings, a scoping review was conducted to map existing evidence and lay the foundation for potential primary research questions. We adopted the Preferred Reporting Items for Systematic Reviews and Meta-Analyses extension for Scoping Reviews (PRISMA-ScR) checklist (Supplementary Appendix 5) and Arksey and O’Malley’s framework as a guide to conduct this scoping review which explores and maps the literature in this field and identifies key concepts, theories, sources of evidence and gaps in the research [17, 18].

### Identifying the research question

The primary research question was: What regulation and governance exists for the implementation and management of PoCT in Australian healthcare settings? With sub-questions including: in what jurisdictions (e.g. state) and clinical contexts (e.g. primary care, hospital, pharmacy etc.) have regulation and governance for the primary review question been used? What are the facilitators and barriers to PoCT implementation that are or could be addressed by governance and/ or regulation? Table 1 shows the Population, Content and Context (PCC) framework used to determine the suitability of the primary research question.

**Table 1:** PCC framework for defining the eligibility of the studies to inform the primary research question

Population	All types of point of care tests used for diagnosis of human disease
Concept	Governance and regulatory guidance for implementation of PoCT including policies, guidelines, frameworks, standards and other information which guides the use of PoCT
Context	Australian healthcare settings including primary care, hospital/ tertiary care, pharmacy, ambulance and community care settings

### Identifying relevant policies, regulation and guidance

We used two strategies to identify relevant national, state and organisational policies and guidance in Australia.

The first strategy involved a search for relevant information through specific government and independent organisation websites and search engines conducted between June and October 2022. This included Google internet search engine, the Australian Government Department of Health and all Australian State and Territory Government Departments of Health and known medical organisations (Supplementary Table 1). Grey literature (information not published commercially) was included to ensure coverage of all evidence and reduce the risk of publication bias [19].

The second strategy was a broad search of published scientific literature conducted using the PubMed database. The search was conducted in October 2022 and updated in December 2023. The search term used was (POCT[Title/Abstract] OR ‘point of care test*’[Title/Abstract] OR ‘bedside test*’[Title/Abstract]) AND (Australia[Title/Abstract]). Abstracts were screened initially against the established inclusion and exclusion criteria (Table 2), then a full review and extraction for remaining articles.

**Table 2:** Inclusion and exclusion criteria

Inclusion criteria	Exclusion criteria
Communicable and non-communicable diseases of human health diagnosed using pathology tests	Self-testing
Describes the regulation or governance in the implementation or management of PoCT in an Australian healthcare setting	Diagnostic imaging PoC tests
	Scientific validation or evaluation of PoC devices
	Testing not provided for patient healthcare

### Data extraction

Data extraction was conducted in two phases. Initially, guided by the eligibility criteria, CK performed the PubMed and grey literature searches in October 2022, with both CK and JP independently screening the identified sources. CK extracted the relevant data, which JP independently verified for accuracy and completeness. Subsequently, JP conducted an additional search in December 2023 to extract any new relevant data. Discrepancies were resolved through discussion between CK and JP.

The information was extracted into a pre-defined template in Microsoft Excel (Version 2410). We extracted the following: author(s), year of publication, source, issue date, context/ study design, aim, setting, jurisdiction, disease, PoC test, regulation/ governance. Additionally, for publications focusing on PoCT implementation, we summarised key findings as implementation facilitators and barriers.

### Thematic analyses

Factors influencing PoCT implementation in identified studies (Supplementary Table 2) were categorised as either barriers or facilitators, aligning with a predefined set of themes: workforce, clinical governance, PoCT workflow, and cost. These codes were iteratively updated as new factors were identified. As we were aiming to describe a body of literature, no risk bias or quality appraisal of the published works were performed in this scoping review, and this would not have impacted the descriptive synthesis of implementation factors. The synthesis of results focused on describing current PoCT guidance and implementation activities in Australia as reported in the literature. This information was tabulated and presented using a narrative approach. Quantitative analysis involved reporting the frequencies of factors identified using Microsoft Excel (Version 2410) for analysis.

## Results

### Selection of sources of evidence

**Fig. 1:**
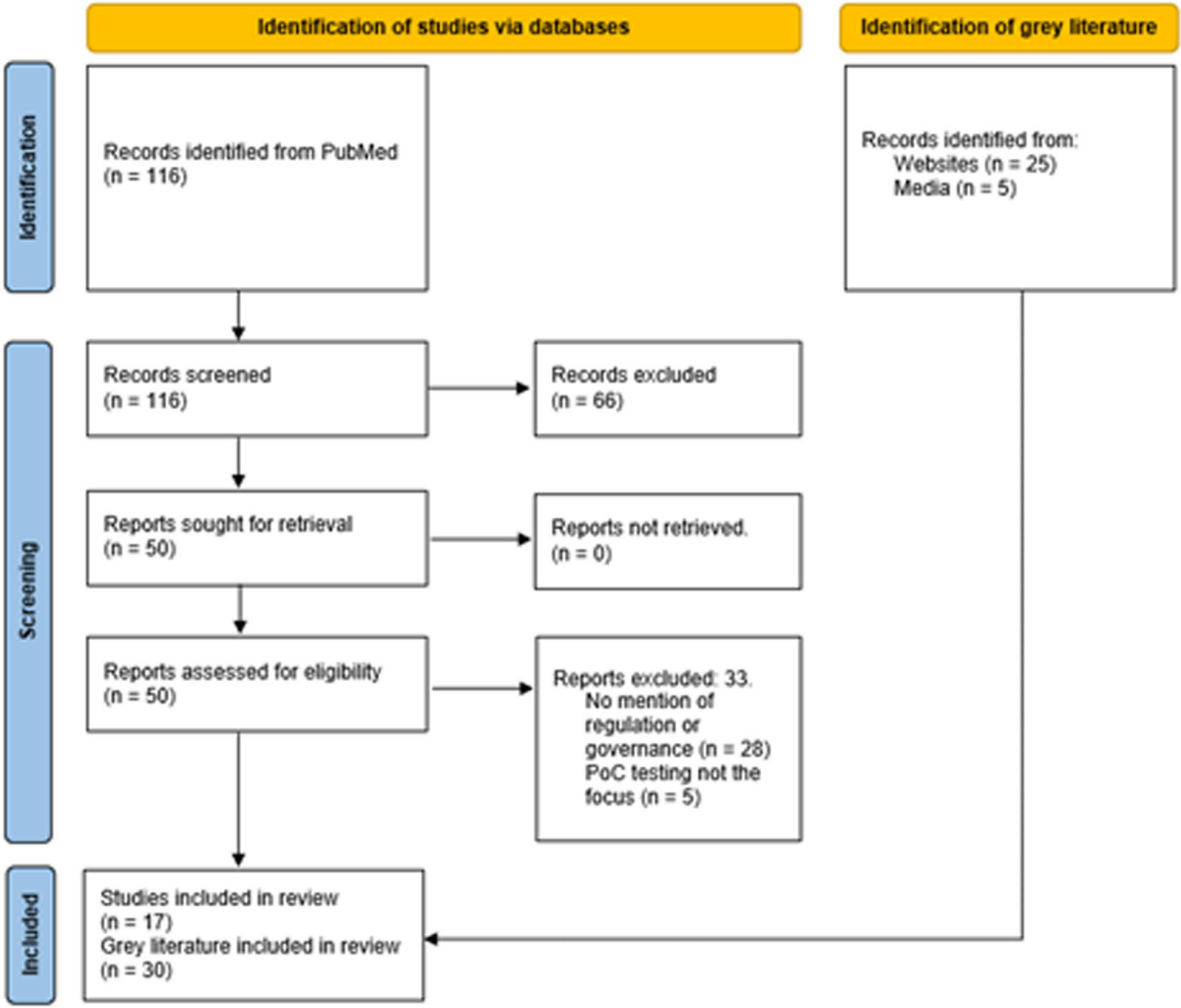
Selection of sources in this review

The PubMed search returned 116 articles which were screened based on their titles and abstracts. Of these, 66 were excluded, the full text of the remaining 50 were examined for applicability, of these, 33 were excluded for not reporting regulation or governance of PoCT. The 17 remaining studies along with 25 regulatory or government documents (standards, guidance, frameworks) and 5 media sources related to governance of PoCT were included in this scoping review (Fig. 1).

### Characteristics of included sources

Sources included four international perspectives, 19 sources provided an Australian national view and 24 focused on jurisdictional levels. The sources included a variety of methodologies such as qualitative and quantitative methods, reports, positions statements, and policies or guidance documents ranging in publication dates from 1999 to 2023. In several cases, there was limited publicly available information regarding the management of PoCT at the jurisdictional level and in individual healthcare settings. Table 3 summarises the characteristics of the 17 included publications that described governance of PoCT in the research. These studies reported various PoC tests with eight targeting infectious diseases (mostly respiratory and sexually transmissible infections (STIs)), four non-communicable diseases and five unspecified diseases applying to PoCT broadly. In the reviewed literature, there was one study that focussed specifically on regulation and governance of PoCT [20]. The remaining publications described or evaluated the implementation of a PoC device which mentioned factors of regulation and governance as barriers and/ or facilitators for PoCT.

**Table 3:** Summary of 17 included publications describing regulation or governance for PoCT in Australian health services

Reference	Setting(s)	Study design	Jurisdiction(s)	Type of PoCT	Regulation/ governance model reported	Aim
**Primary care**						
Saha et al. [21]	General Practice (GP); Rural & remote	Evaluation study	QLD, NSW, SA, WA, NSW, Vic	Infectious diseases: STIs, COVID-19	Connectivity system compliant with Australian digital health regulatory guidelines	To design and deploy connectivity solutions for primary care in remote and regional Australia and evaluate the uptake, effectiveness, and impact on programmatic implementation
Lafferty et al. [22]	General Practice; Rural & remote	Qualitative study	SA, WA, NT and QLD	Infectious diseases: STIs	Research study, network governance approach	To identify barriers and facilitators to scaling up STI PoCT in remote Aboriginal communities within Australia
Lingervelder et al. [23]	General Practice	Expert review	Australia, England, Norway, Netherlands	Unspecified	N/A, refers to Australian NPAAC and RACGP standards	To identify the current value networks in place applying to PoCT implementation at general practices in England, Australia, Norway and the Netherlands that support the successful implementation, sustainability and scale-up of PoCT
Gunnarsson et al. [24]	General Practice	Observational study	Queensland	Infectious diseases: Group A streptococci	Research study within usual clinical governance	To clarify to what extent medical practitioners’ decisions to prescribe antibiotics are changed by a PoC test
Mullens et al. [25]	Community care; Mobile; Rural & remote	Mixed methods study	Queensland	Infectious diseases: STIs	Research study	To establish and evaluate the implementation of a mobile clinic van as a method to engage individuals in regional areas with anonymous HIV/ STI PoCT
Spaeth et al. [26]	General Practice; Rural & remote	Evaluation; economic study	Northern Territory	Non-infectious diseases	NT PoCT Program	To determine the cost-effectiveness of utilising PoCT to aid decisions regarding the emergency medical retrievals of patients at remote health centres in the Northern Territory
Tirimacco et al. [27]	General practice	Observational study	SA, NSW, Vic	Non-infectious diseases	Research program based on RACGP (Interim Standards)	To describe the development and evaluation of an accreditation program for PoCT in general practice
Shephard and Gill [28]	Community care; Rural & remote	Case study	Australia wide	Non-infectious diseases: diabetes	QAAMS program; RCPA QAP providing QC/QA	Case study to demonstrate the quality-assured conduct of PoCT to assist diabetes management in over 110 Aboriginal and Torres Strait Islander medical services across Australia
Tirimacco [20]	General practice	Expert review	Australia wide	Unspecified	References ISO15189, ISO22870, NPAAC	To discuss the background to development of interim standards for general practice PoCT trial
Ballard et al. [29]	Community care; Mobile; Rural & remote	Observational study	Victoria	Infectious diseases: COVID-19	Accredited by NATA in alignment with laboratory accreditation	To describe the process for implementation of a mobile LabVan for COVID-19 testing
Hengel et al. [9]	General Practice; Rural & remote	Case study	Australia wide	Infectious diseases: COVID-19	Aligned with NPAAC standards with networked, community led governance	To describe an implementation framework for decentralised PoCT in Aboriginal and Torres Strait Islander communities
Shephard et al. [30]	General Practice; Rural & remote	Expert review	Australia wide	Unspecified	N/A, discussed existing regulation and governance models	To explore the clinical, operational, cultural, and cost benefits of PoCT in the Australian rural and remote health sector and describe some of the current challenges and limitations of this technology
**Hospital**						
Muhi et al. [10]	Emergency Department (ED)	Cohort study	Victoria	Infectious diseases: COVID-19	Research study within usual clinical governance	To determine performance characteristics and identify the challenges and a framework for implementation of rapid antigen tests in a low-prevalence setting
Li et al. [31]	Emergency Department; Rural & remote	Observational study	New South Wales	Non-infectious diseases: circulatory system illnesses	NSW Health Pathology Managed PoCT service	To evaluate the impact of PoCT on patient outcomes in rural and remote EDs in NSW
Li et al. [32]	Emergency Department; Rural & remote	Observational study	New South Wales	Unspecified	NSW Health Pathology Managed PoCT service	To evaluate PoCT data linkage, integration and quality in NSW PoCT data system and their impact on patient care for monitoring of PoCT service
Dahm et al. [33]	Emergency Department; Rural & remote	Qualitative study	New South Wales	Unspecified	NSW Health Pathology Managed PoCT service	To understand from frontline clinical staff the operational impact of the implementation of PoCT across rural and remote EDs in NSW
Turner et al. [34]	Emergency Department	Observational study	Tasmania	Infectious diseases: Influenza	Research study within usual clinical governance	To pilot sentinel syndromic surveillance for influenza-like illness in Tasmania

**Fig. 2:**
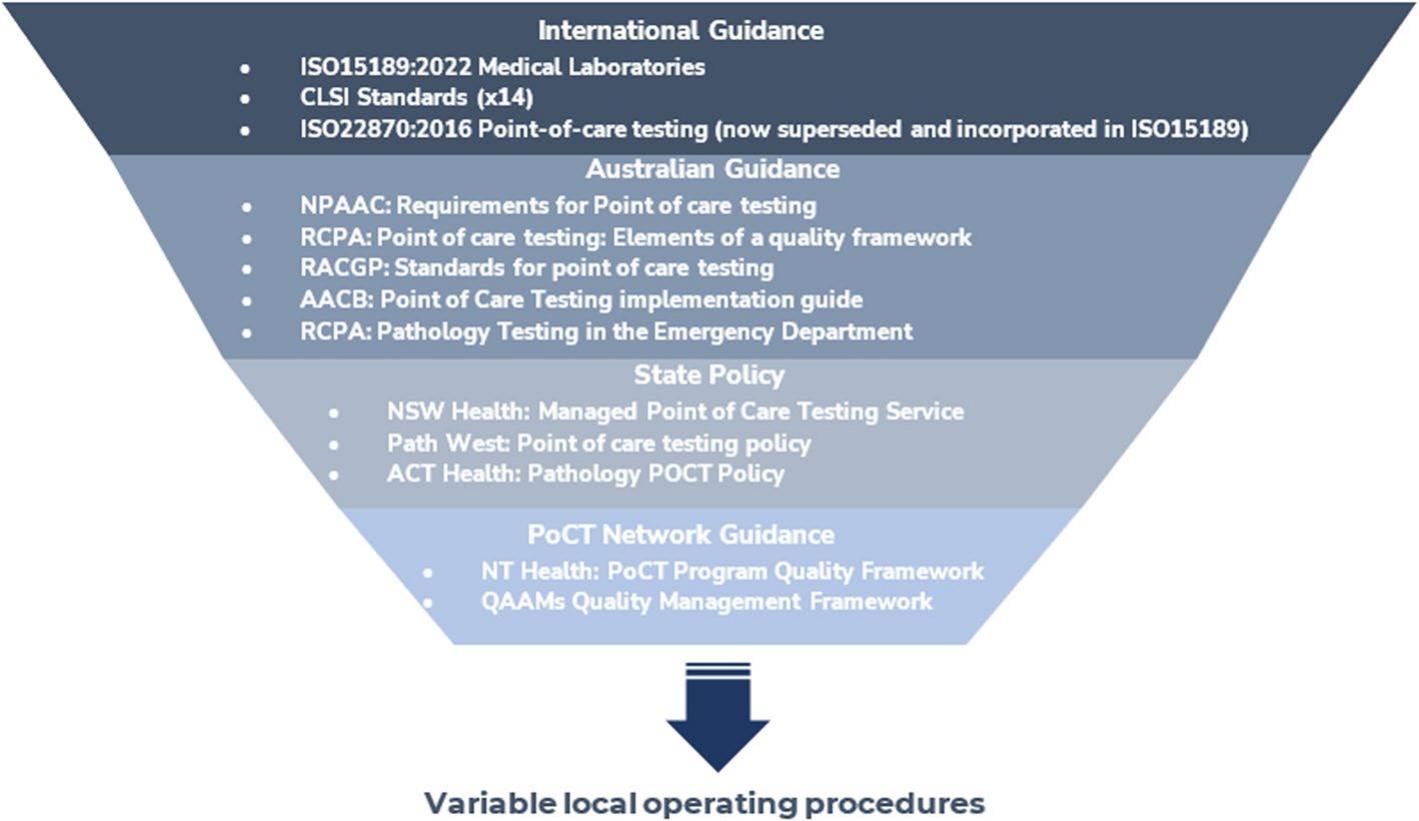
Summary of standards and guidance which apply to the use of PoCT in Australia at various levels without mandated adoption (summary of Supplementary Table 3)

**Fig. 3:**
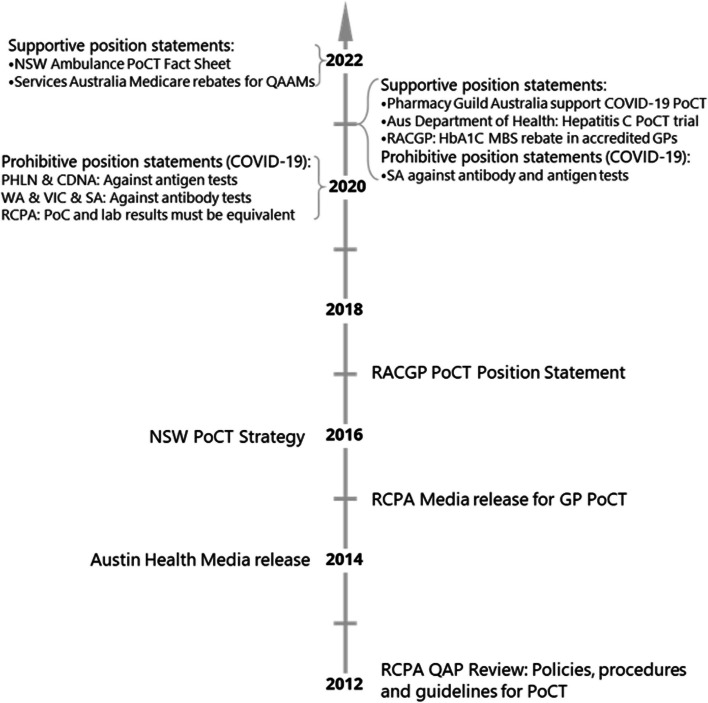
Timeline of grey literature relating to PoCT in Australia (summary of Supplementary Table 4)

Fifteen current PoCT governance documents including six standards, five guidelines and four frameworks with three in operation internationally and six in Australian jurisdictions were identified (Fig. 2, Supplementary Table 3) [3, 13, 14, 35–46]. The remaining grey literature (Fig. 3, Supplementary Table 4) included 15 publications addressing the use of PoCT in Australia with four media articles, seven position statements and a guideline, fact sheet, strategy and review [11, 47–60].

### Jurisdictions and clinical contexts for the implementation and management of PoCT in healthcare

Evidence of PoCT was found in each jurisdiction (states and territories) of Australia. The reviewed grey literature (Supplementary Table 3) included three sources at the international level, 15 sources at the national Australian level, and 12 at a jurisdictional level with three in New South Wales (NSW) and Victoria (VIC), the most populous states in Australia followed by two in Western Australia (WA), one in the Northern Territory (NT), the Australian Capital Territory (ACT), South Australia (SA), and Queensland (QLD). There were no included grey literature sources from Tasmania.

Published studies (Table 3) included Australian-wide settings and all Australian states and territories, except for the Australian Capital Territory. Twelve of the 17 studies described PoCT in primary care settings and five within hospital settings. The most represented clinical context in the literature was rural and remote settings as 11/17 studies referenced PoCT use outside metropolitan areas of Australia, and two of these studies used mobile laboratories to deliver PoCT. There were no included studies in the settings of pharmacy and ambulance although grey literature was identified in these areas.

### Facilitators and barriers to PoCT implementation

**Fig. 4:**
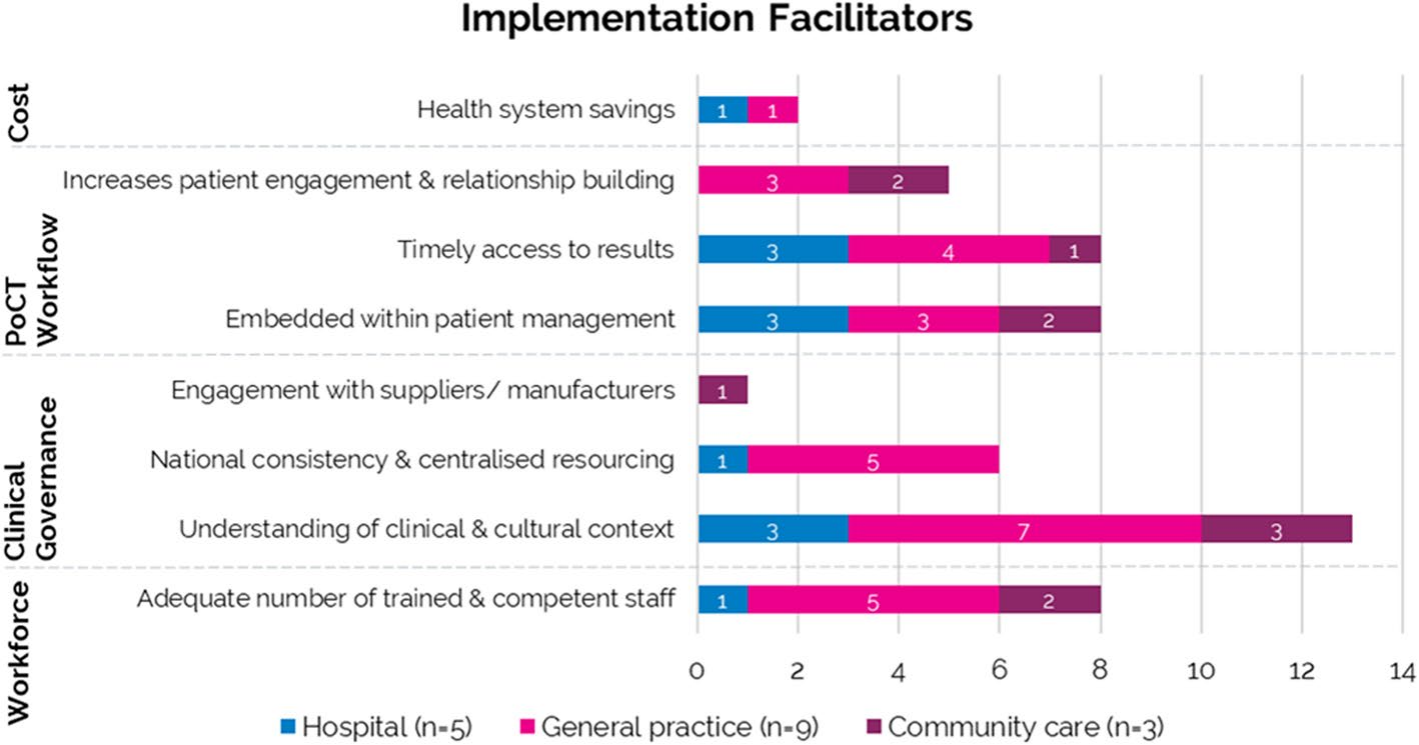
Summary of facilitators for the implementation of PoCT

**Fig. 5:**
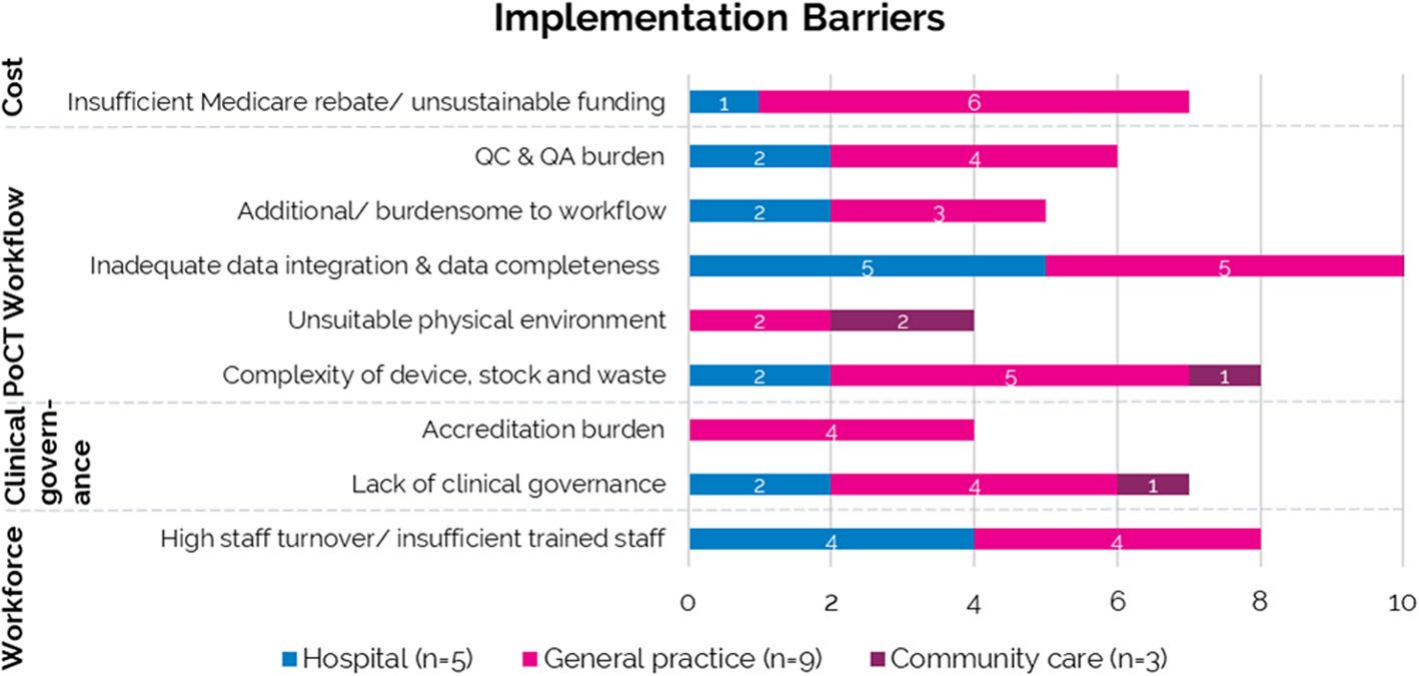
Summary of barriers for the implementation of PoCT

Factors relevant to the implementation of PoCT reported in the literature were summarised under four themes: cost, PoCT workflow, clinical governance, and workforce (Figs. 4 and 5). Only one study mentioned accreditation by NATA [29], although four reported alignment or compliance with the NPAAC or Royal Australian College of General Practitioners (RACGP) guidelines [9, 20, 23, 27]. Most were conducted under a research governance framework and did not mention if these were compliant with national regulatory guidance. The implementation facilitator most frequently reported was an understanding of clinical and cultural context for PoCT (Fig. 4). The most reported barrier to implementation was data management, with 10/17 studies reporting inadequate data integration and data completeness from PoC test results (Fig. 5).

## Discussion

This scoping review aimed to elucidate the current state of point-of-care testing (PoCT) regulation and governance within Australian healthcare services. Although several national standards were identified, their application is not mandatory, except within the contexts of laboratory accreditation frameworks or specific state and program-based policies. It is noteworthy that accreditation for PoCT remains optional for Australian healthcare services that do not receive Medicare reimbursement for these tests. Despite this, healthcare services have continued to deploy PoCT, both within and beyond the bounds of national regulatory guidance, demonstrating a lack of uniform adoption across Australian health services. The research concerning the implementation of PoCT in accredited healthcare settings was sparse; instead, numerous publications concentrated on the creation of governance models tailored to specific programs. This indicates that current standards and accreditation mechanisms may be inadequate for certain clinical scenarios and could benefit from revisions to encourage the standardised adoption of PoCT. Research spanning various healthcare sectors highlighted a range of facilitators and barriers to the implementation of PoCT, indicating the need for further guidance on its practical application in Australian healthcare environments.

This Australian review is consistent with experiences from other high-income countries, reflecting the importance of PoCT to supplement traditional, laboratory based diagnostic testing. Shared challenges in clinical governance, quality assurance, workforce training and data connectivity were reported, potentially hindering widespread adoption of accredited PoCT [61–64]. The understanding of cultural and clinical contexts, a critical component of clinical governance, was reported in 13 out of 17 Australian studies as a key facilitator to implementation. An alternative model demonstrating successful widespread implementation of PoCT is Norway’s dedicated organisation, NOKLUS. They provide pathology support for PoCT services nationwide, overseeing all PoCT in primary care, offering support and quality management, and enabling national level reporting of PoCT usage tailored to specific geographical contexts [23].

Most studies identified in this review described the use of PoCT in non-metropolitan areas of Australia. In Australian rural and remote settings, limited access to healthcare, specialised staff, and laboratory infrastructure poses challenges, and the effective use of PoCT has been demonstrated as a viable solution for improving access to diagnostics and may be applicable to other underserved settings globally [30, 33, 65]. This finding is significant as it demonstrates that rural and remote settings have been early adopters and leaders in PoCT implementation, although not all facilitators and barriers in these settings may be applicable to larger scale and more resourced metropolitan settings. For example, an unsuitable physical environment with unreliable internet access posed a barrier to PoCT in these settings including mobile testing programs [22, 29, 66, 67]. In this review no guidance was found specific to PoCT use in rural or remote settings, despite this being the setting where PoCT can provide the greatest benefit. However, the barriers described in the identified studies, including staffing and infrastructure challenges may make it harder for less resourced settings to comply with the existing regulation compared to larger metropolitan healthcare services.

Several studies have demonstrated that overcoming barriers to PoCT implementation can provide clear benefits, such as reducing overall healthcare costs, improving patient outcomes in rural and remote areas, and addressing inequalities in healthcare access for Indigenous communities. The potential cost-effectiveness of PoCT at the healthcare sector level, despite higher per-test costs, was demonstrated through reduced time to results and faster connection to care [68, 69]. The majority of primary care settings (6/9), including rural and remote services, reported unsustainable funding as a barrier to PoCT implementation despite the reported benefits justifying investment in PoCT infrastructure [26, 30, 70]. There is a need for investment in PoCT to cover implementation costs and ongoing expenses, including workforce training, data connectivity, test reagents, and accreditation to enable scale-up of PoCT [22].

While the NPAAC guidelines establish a requirement for a designated person accountable for PoCT, several studies highlighted the benefits of centralising some governance functions to support PoCT, alleviating the burden of regulation and PoCT accreditation on individuals [9, 46, 49, 60, 70–72]. These findings were consistent with the guidance from The Australasian Association of Clinical Biochemists and the Royal College of Pathologists Australasia (RCPA) which recommend an enhanced governance framework under a central PoCT committee, with decisions made under pathologists’ stewardship to ensure the safety, quality, and utility of PoC tests [38, 45]. Similarly, guidelines in the UK and USA recommend interdisciplinary committees to oversee the clinical governance of PoCT. These committees, compromising laboratory staff, clinicians, nurses, IT and operational support staff are seen as important for helping laboratory staff better understand clinical needs, aligning with our finding of the understanding the clinical and cultural context being the most reported facilitator for PoCT implementation [73, 74]. To ensure the effectiveness of PoCT, having a sufficient number of trained and competent staff is critical. Workforce was reported as both a facilitator and barrier for PoCT implementation. In hospital settings, inadequate trained staff indicated a need for greater collaboration between pathology laboratories and patient-interface departments such as emergency departments and intensive care wards. Similarly, primary care PoCT programs noted challenges in retaining trained staff with high turnover rates [22, 33, 66]. The administrative and time burden on non-laboratory trained staff to manage PoCT quality and compliance with regulations designed primarily for laboratories was a reported barrier to PoCT. Streamlining workforce support through shared training, templates, resources and expert advice could alleviate burdens on individual health services and facilitate PoCT implementation across healthcare settings.

Evidence from this review highlighted data connectivity and integration with existing medical systems as a substantial challenge, reported as a barrier in ten studies (10/17) across all settings [32, 75]. The lack of standardisation in proprietary software for most PoC devices complicates the flow of data and reporting of results, impeding the introduction of new systems into the existing complex digital infrastructure including laboratory information systems and electronic health records [21]. However, addressing this challenge is crucial, as effective PoCT necessitates a robust method for recording results to ensure availability of test history for patient care teams and public health investigations. Despite these challenges, integration offers potential efficiency gains including centralised reporting, remote management, streamlined clinical communication, and longitudinal monitoring and surveillance, all which could facilitate embedding PoCT within clinical workflows for widespread implementation [21]. The rapid pace of technological innovations in PoC diagnostics poses challenges for regulatory bodies overseeing these devices and their data management systems. As new PoCT technologies emerge, regulators must ensure they meet stringent standards for patient safety, privacy and data security. Moreover, governance frameworks for PoCT implementation must evolve to address interoperability with existing healthcare infrastructure, enabling complete patient health records that capture results from both PoC and laboratory-based tests.

This scoping review highlights the lack of standardised PoCT governance frameworks, with many being jurisdiction or setting specific despite national accreditation programs. Additionally, the need for understanding local clinical and cultural contexts, identified as a key PoCT facilitator, suggests challenges in implementing a standardised approach. However, a limitation of this review is that it does not explore the reasons behind the low adoption rate of PoCT accreditation among Australian healthcare services. It remains unclear how PoCT should be regulated, accredited and funded in Australia with current variability in governance models and adherence to national standards. Future research should investigate the reasons for this low adoption rate of PoCT accreditation among Australian healthcare services and seek insights from providers who have successfully implemented accredited PoCT services. These insights could inform potential regulatory changes to facilitate broader implementation. Our findings provide a basis to establish appropriate guidance for practical PoCT use across different healthcare settings and evaluate how additional guidance or regulation impacts clinical outcomes. Such studies could assess the effect of various PoCT governance models on patient outcomes, healthcare effectiveness, and cost–benefit analyses, as well as qualitative research on patient and healthcare worker perspectives. To achieve a comprehensive understanding of the objectives, risks, and opportunities for PoCT guidance, future research should engage key Australian organisations involved in PoCT delivery, including peak bodies for medical scientists, pathologists, and patient-centred care advocates. Governments could consider minimising regulatory issues and reducing barriers described by healthcare providers to realise the potential benefits of PoCT.

This review provided a comprehensive mapping of evidence on regulation and governance on PoCT in Australia and to our knowledge the first to combine sources from research, governing bodies and media publications. The strengths of this scoping review are the inclusion of grey literature to capture the policies and positions of various organisations regarding PoCT in Australia. We consolidated this information across healthcare settings, often reviewed in isolation, which in future could help to understand the specific needs of services. While we report few studies that used PoCT in an accredited environment, it is likely this is occurring and not being reported in the research or medical media domain and therefore not captured in this review. Future research could consider case studies of these sites to develop best practice and provide guidance on implementing accrediting PoCT services.

## Conclusion

In conclusion, this review highlights a significant gap in published research on regulatory and governance frameworks for PoCT in Australia, demonstrating a highly variable landscape. Clinical governance is crucial for the effective availability and use of PoCT, which in turn improves and democratises access to quality healthcare. Successful PoCT programs in Australia have demonstrated diverse implementation models, ranging from locally managed to decentralised and independently governed clinical governance and quality management systems. The minimal adoption of accreditation highlights the need to reassess regulatory pathways for PoCT to ensure sustainable funding and patient benefits. A streamlined approach to PoCT oversight, recognising the distinct challenges in non-traditional laboratory settings, is essential for maintaining high-quality results and meeting patient and clinician needs.

To leverage the potential of PoCT technology, governments should consider implementing a clear regulatory framework. This framework should cover test selection, reimbursement, integration of PoCT results with medical records, continuous workforce training, and quality assurance protocols in coordination with laboratories. Effective regulation and governance can enhance PoCT usage in diverse healthcare environments, thereby expanding patient access to pathology services and improving healthcare quality in Australia.

## Supplementary Information

Supplementary Material 1.

## Data Availability

No datasets were generated or analysed during the current study.

## Abbreviations

ED: Emergency Department
GP: General Practice
ISO: International Organization for Standardization
NPAAC: National Pathology Accreditation Advisory Council
PoC: Point of care
PoCT: Point-of-care testing
QA: Quality Assurance
QC: Quality Control
STI: Sexually Transmissible Infection
