# Supplementary material for: Regulation and governance for the implementation and management of point-of-care testing in Australia: a scoping review
Source: BMC Public Health. 2025 Feb 24;25:758. doi: 10.1186/s12889-025-21894-2 (PMC11849271; doi:10.1186/s12889-025-21894-2)
Supplement: Supplementary file 1 — Supplementary Material 1. [file 12889_2025_21894_MOESM1_ESM.docx]

**Regulation and governance for the implementation and management of point-of-care testing in Australia: a scoping review**

**Supplementary appendices**

Supplementary Table 1. Targeted website searches for sourcing relevant grey literature.

| **Organisation** | **Website** |
| --- | --- |
| Australian Government Department of Health and Aged Care | https://www.health.gov.au |
| Victorian Government Department of Health | https://www.vic.gov.au/department-health |
| New South Wales Health | https://www.health.nsw.gov.au |
| South Australia Health | https://www.sahealth.sa.gov.au/wps/wcm/connect/public+content/sa+health+internet |
| Northern Territory Health | https://health.nt.gov.au |
| Western Australia Government Department of Health | https://ww2.health.wa.gov.au |
| Queensland Government Health | https://www.health.qld.gov.au |
| Tasmanian Government Department of Health | https://www.health.tas.gov.au |
| Australian Capital Territory Department of Health | https://www.health.act.gov.au/ |
| Alfred Health Pathology | https://www.alfredhealth.org.au/services/pathology |
| Austin Pathology | https://www.austinpathology.org.au/test-directory/1711 |
| Eastern Health Pathology | https://www.easternhealth.org.au/about-us/item/363-pathology |
| Goulburn Valley Health Pathology | http://www.gvhealth.org.au/health-professionals/diagnostic-services/pathology/ |
| Monash Health Pathology | https://monashpathology.org |
| Northern Pathology | https://northernpath.org.au |
| Peter Mac Pathology | https://www.petermac.org/services/diagnosis-investigations/pathology |
| Royal Children's Hospital Melbourne / Royal Women's Hospital Laboratory Services | https://www.rch.org.au/labservices/ |
| Royal Melbourne Hospital Pathology | https://www.thermh.org.au/health-professionals/clinical-services/pathology |
| St Vincent’s Pathology Melbourne | https://path.svhm.org.au/ |
| Victorian Cytology Service Pathology | https://www.vcs.org.au/pathology/ |
| Australasian Association for Clinical Biochemistry and Laboratory Medicine (AACB) | https://www.aacb.asn.au |
| Australian College of Rural and remote medicine (ACRRM) | https://www.acrrm.org.au/ |
| Australian Point-of-care Practitioners Network (APPN) | https://www.appn.net.au |
| Flinders University International Centre for Point of Care Testing (ICPOCT) | https://www.flinders.edu.au/international-centre-for-point-of-care-testing |
| Integrated Cardiovascular Clinical Network South Australia (iCCNet) | https://www.iccnetsa.org.au |
| National Association of Testing Authorities (NATA) | https://nata.com.au/ |
| National Pathology Accreditation Advisory Council (NPAAC) | https://www1.health.gov.au/internet/main/publishing.nsf/Content/health-npaac-index.htm |
| Northern Territory PoCT Network | https://ntpoct.com.au/ |
| Quality Assurance for Aboriginal and Torres Strait Islander Medical Services (QAAMS) | https://qaams.org.au |
| Royal Australian College of General Practitioners (RACGP) | https://www.racgp.org.au |
| Royal College of Pathologists of Australasia (RCPA) | https://www.rcpa.edu.au/Home |
| Test, Treat and Go Program (TANGO2) | https://www.ttango.com.au |

Supplementary Table 2. Definitions of implementation facilitators and barriers as evidenced by available literature.

| **Theme** | **Facilitator** | **Defined as** |
| --- | --- | --- |
| **Workforce** | Adequate number of trained & competent staff | - Multiple staff trained in PoCT at facility - Continuing education, training and competency assessments provided for staff - Skilled trainers able to communicate laboratory concepts to non-laboratory staff (e.g. QC and QA) |
| **Clinical Governance** | Understanding of clinical & cultural context | - Clear clinical purpose for PoCT in a defined population e.g. COVID-19 screening risk for hospital triage; targeted antimicrobial prescriptions based upon PoCT results rather than clinical presentation alone - Staff knowledge of population disease burden and aware of how PoCT can improve patient management and reduce loss to follow up which is communicated to staff and patients - Established pathways for confirmatory testing through pathology services - Supportive medical leadership with acknowledgement of the change in practice for staff performing PoCT - Peer testing operators that understand the cultural context of the disease impact on patients |
|  | National consistency & centralised resourcing | - Single authority on specific PoC tests with consistent guidelines - Centralised resources available for PoCT e.g. standard operating procedures, instructions, forms and documents relating to training, quality control (QC) and quality assurance (QA) - Centralised support available for PoCT e.g. phone line for technical troubleshooting, result analysis - Education on PoCT within healthcare practice - Clinical networks comprised of practitioners that operate the same PoC device for support and information sharing |
|  | Engagement with suppliers/ manufacturers | - Support services and training available for PoC device operation provided by suppliers and manufacturers |
| **PoCT Workflow** | Embedded within patient management | - PoCT implemented routinely into clinical workflow with staff aware of the process and how to operate the PoC device - PoCT integrated into the health service clinical framework and used as part of the diagnostic protocol not a standalone tool - Clinically effective PoCT that results in action for patient management and treatment |
|  | Timely access to results | - Results enable same day diagnosis and treatment reducing patient loss to follow-up - Accurate reporting of results and recording of data in existing systems including for program evaluation - PoCT more convenient than waiting for laboratory services and results - Timely results prevent of overcrowding of hospital emergency departments and facilitate patient referral with increased information available |
|  | Increases patient engagement & relationship building | - PoCT creates an additional opportunity to discuss health with patient and create trust between patient and healthcare practitioner - More information available for patient management increasing satisfaction of patients and healthcare practitioners - Patient interest in PoCT and subsequent engagement in their healthcare - Patient acceptability of results from rapid tests - Patient acceptability of less invasive sample types e.g. finger prick blood sample for PoCT versus venous blood for laboratory testing |
| **Cost** | Health system savings | - Prevention of unnecessary medical evacuations from remote areas and reduction of patient transfers in rural areas reduces system costs - Medicare funding for PoCT (available for some tests) - Evidence of cost-effectiveness for specific PoC tests and diseases |
|  |  |  |
|  |  |  |
| **Theme** | **Barrier** | **Defined as** |
| **Workforce** | High staff turnover/ insufficient trained staff | - Staff turnover is high (especially with locum staff in remote areas) resulting in high burden to maintain trained staff able to perform PoCT - Low confidence in staff interpretation of results limiting clinical action from PoC test results - Infrequent use of PoC device results in lack of staff confidence in performing PoCT - Face to face training too costly and impractical for rural & remote settings to provide equivalent training to urban settings |
| **Clinical Governance** | Lack of clinical governance | - Lack of recommendations for PoCT in clinical practice guidelines - Ineffective communication between organisations about PoC tests being used e.g. primary care referrals to hospitals based upon results - Lack of awareness of PoCT being offered in services for patients and staff in services with high turnover |
|  | Accreditation burden | - NATA accreditation burdensome for clinics where practitioners are not supported or resourced to meet current standards - Accreditation not adapted to PoCT settings which cannot meet the same standards as laboratories |
| **PoCT Workflow** | Complexity of device, stock and waste | - Challenges or complexity in using the device, performing the test, entering results in the system - Issues with the PoC device hardware and test components - Insufficient PoC devices relative to demand for PoCT - Generation of large amounts of waste from device and test components - Stock levels difficult to manage with demand and expiry dates leading to wastage |
|  | Unsuitable physical environment | - Lack of dedicated, suitable space for PoC device and testing - Issues with power supply & temperature control - Lack of storage space available to maintain reagent stock levels |
|  | Inadequate data integration & data completeness | - Lack of real-time data integration with PoCT and patient record system - Multiple software required to be used with duplication of data entry with PoCT specific systems - Limited PoCT results recorded in Laboratory information management systems and electronic medical records for evaluation of programs - Issues with data quality and completeness |
|  | Additional/ burdensome to workflow | - Existing high workloads of staff limiting PoCT use to non-peak times - Difficult to obtain staff and/ or management buy-in and engagement for additional work - PoCT not embedded within workflow and seen as an additional burden - Insufficient staff time to manually record PoCT results and PoC device information - PoCT result interpretation out of scope of staff (nurse, healthcare assistant) duties |
|  | Quality control (QC) and quality assurance (QA) burden | - Insufficient documentation and communication to staff regarding QC and QA - Burden to perform QC and QA - Forgetting to perform QC checks - Lack of provided QC or QA material from supplier |
| **Cost** | Insufficient Medicare rebate/ unsustainable funding | - Cost of PoCT not matched by MBS rebate - PoCT more expensive than equivalent laboratory testing - Costs additional to instrument and reagents e.g. QC, QA, training, IT for system integration not sufficiently covered by MBS rebate or program costs - High cost of accreditation and ongoing QA - Lack of funding incentives to perform PoCT |

Supplementary Table 3. A summary of current PoCT standards, guidelines and framework documents identified in the public domain and reviewed for this report that address PoCT in Australia.

| **Year** | **Author** | **Region** | **Type** | **Application** | **Title** |
| --- | --- | --- | --- | --- | --- |
| 2023 (35) | Clinical and Laboratory Standards Institute (CLSI) | International | Standards | CLSI publishes standards and guidelines to promote global laboratory standards in a variety of ‘Speciality Areas’ which includes ‘Point-of-Care-Testing’. | As of December 2023, CLSI has 15 published standards and guidelines which define or are associated with laboratory and PoCT quality procedures |
| 2022 (13) | International Organisation for Standardisation (ISO) | International | Standards | International standard for medical laboratory testing. | *ISO 15189:2022 Medical laboratories — Requirements for quality and competence* |
| 2021 (3) | National Pathology Accreditation Advisory Council (NPAAC) | Australia | Standard | NATA accredited medical laboratory services | *Requirements for Point of care testing, 2^nd^ ed.* |
| 2020 (36) | Queensland Health | Queensland | Guideline | Guidelines for COVID-19 PoCT for Indigenous Australians. | *Aboriginal and Torres Strait Islander COVID-19 Point-of-Care Testing Program Guideline* |
| 2019 (37) | Ambulance Victoria | Victoria | Guideline | Indicates that PoCT for blood glucose is routinely conducted by paramedics. | *Clinical Practice Guidelines for Ambulance and MICA Paramedics* |
| 2019 (38) | Australasian Association of Clinical Biochemists (AACB) | Australia | Standard | Health services with biochemistry based tests. | *Point of Care Testing implementation guide* |
| 2018 (39) | Australasian College for Emergency Medicine & Royal College of Pathologists of Australasia | Australia | Guideline | Guidance on pathology requesting and collecting in emergency departments (EDs), this encompasses all pathology testing and includes one section on PoCT recommending PoCT decisions be joint with a pathology laboratory. | *Pathology Testing in the Emergency Department* |
| 2018 (40) | Royal Australian College of General Practitioners (RACGP) | Australia | Standards | Accredited ‘GP clinics that perform PoCT’ | *Standards for point of care testing, 5th ed.* |
| 2018 (41) | NSW Health | NSW | Framework | State-wide framework within NPAAC standards. | *Policy Directive: Managed Point of Care Testing (PoCT) Service* |
| 2017 (42) | Path West | WA | Guideline | Use of PoCT across WA Health services. | *PathWest Point-of-care testing policy (POL-249)* |
| 2016 (14) | International Organisation for Standardisation (ISO) | International | Standards | International standard for PoCT use in healthcare facilities, required for accreditation. Now incorporated into ISO15189:2022. | *ISO 22870:2016 Point-of-care testing (POCT) — Requirements for quality and competence* |
| 2015 (43) | ACT Health | ACT | Guideline | Use of PoCT for pathology across ACT Health services. | *Pathology Point-of-care Testing (PoCT) Policy* |
| 2015 (44) | NT Health & Flinders ICPOCT | NT | Framework | Specifically for the use of PoCT devices (i-STAT initially) in a dedicated program run to meet challenges in remote settings with high prevalence of cardiac and renal disease. | *NT POCT Program Quality Framework and Initiatives* |
| 2014 (45) | Royal College of Pathologists Australasia (RCPA) | Australia | Framework | Medical pathology services (& other PoCT services). | *Point of care testing: Elements of a quality framework* |
| Since 1999 (46) | Flinders ICPOCT & RCPA Quality Assurance Programs | Australia | Framework | Specifically for sites participating in the Quality Assurance for Aboriginal and Torres Strait Islander Medical Services (QAAMS) program, required to receive Medicare rebates for PoCT services for Aboriginal and Torres Strait Islander peoples. | *QAAMs quality management framework* |

Supplementary Table 4. A summary of position statements, media and other grey literature identified in the public domain and reviewed for this report that address PoCT in Australia.

| **Year** | **Author** | **Region** | **Type** | **Application** | **Title** |
| --- | --- | --- | --- | --- | --- |
| 2022 (47) | Services Australia | Australia | Guideline | Explains Medicare rebates for PoCT including the QAAMS service for Indigenous health. | *Your guide to Medicare for Indigenous health services* |
| 2022 (48) | NSW Ambulance | NSW | Fact sheet | PoCT by NSW Ambulance designed to provide the right care at the right time in the right place which ultimately has a positive impact for patients and on ED demand within NSW. | *Point of Care Testing (PoCT) in NSW Ambulance* |
| 2021 (11) | RACGP | Australia | News/ media | Update on the inclusion of glycated haemoglobin (HbA1c) as a Medicare rebated test if a GP practice is accredited for PoCT. | *MBS point-of-care testing: What GPs need to know* |
| 2021 (49) | Australian Government Department of Health and Aged Care | Australia | News/ media | Announcement of a new PoCT trial for Hepatitis C, aligned with the national Hepatitis C policy. | *Improving diagnosis for hepatitis C patients* |
| 2021 (50) | The Pharmacy Guild of Australia | Australia | Position statement | Supports community pharmacies accessing and selling PoC tests that are approved by the TGA. | *Position Statement COVID-19 Point-of-Care Testing* |
| 2021 (51) | SA Department of Health | South Australia | Position statement | Statements prohibiting the use of SARS-CoV-2 rapid antibody and antigen tests. | *Emergency Management (Prohibition of certain Point of care tests)* |
| 2020 (52) | RCPA | Australia | Position statement | The results of PoC tests either must be equivalent in quality and accuracy to those from an accredited pathology laboratory, or show a demonstrable benefit to patient care that exceeds the risks; used only in appropriate and select circumstances; subject to governance and quality systems. | *Point of care testing.* |
| 2020 (53) | Victorian Department of Health and Human Services | Victoria | Position statement | Against use of rapid antigen tests to diagnose SARS-CoV-2 infection outside of a research framework, or unless specifically advised by the Department of Health. | *Position Statement on rapid antigen tests for diagnosis of SARS-CoV-2 in symptomatic persons* |
| 2020 (54) | Government of Western Australia Department of Health | Western Australia | Position statement | Prohibition of PoC antibody tests for SARS-CoV-2. | *Prohibition on the Use of Point of Care Serology Tests Directions* |
| 2020 (55) | Public Health Laboratory Network and Communicable Disease Network of Australia | Australia | Position statement | Statements advising against SARS-CoV-2 Rapid Antigen Tests during the COVID-19 pandemic. | *Joint Statement on SARS-CoV-2 Rapid Antigen Tests* |
| 2017 (60) | RACGP | Australia | Position statement | Evidence based PoCT should be accessible via general practice through Medicare, and unnecessary regulatory barriers to its adoption in general practice should be removed. | *Point of Care Testing Position Statement* |
| 2016 (56) | NSW Health Pathology | NSW | Strategy | Strategy for PoCT in NSW to clearly define the service model and consolidate and support the service. | *Point of Care Testing (PoCT) Strategic Plan 2016-2018* |
| 2015 (57) | RCPA | Australia | News article | Overview of PoC tests with case studies for GPs, highlighting the requirements for clinical oversight, governance, quality control, staff training and competence. | *Common Sense Pathology, a regular case-based series on practical pathology for GPs: Point of Care Testing* |
| 2014 (58) | Austin Health | Victoria | Media statement | Consolidation with regional labs throughout Victoria and the expansion of PoC Testing with Austin Health oversight. | *Big changes for Austin Health Pathology* |
| 2012 (59) | RCPA Quality Assurance Programs | Australia | Review | Overview of policies, procedures and guidelines for PoCT in 2011 including comparisons with other countries. | *Review: Policies, procedures and guidelines for point-of-care testing* |

Supplementary Table 5. Preferred Reporting Items for Systematic reviews and Meta-Analyses extension for Scoping Reviews (PRISMA-ScR) Checklist

| **SECTION** | **ITEM** | **PRISMA-ScR CHECKLIST ITEM** | **REPORTED ON PAGE #** |
| --- | --- | --- | --- |
| **TITLE** | | | |
| Title | 1 | Identify the report as a scoping review. | 1 |
| **ABSTRACT** | | | |
| Structured summary | 2 | Provide a structured summary that includes (as applicable): background, objectives, eligibility criteria, sources of evidence, charting methods, results, and conclusions that relate to the review questions and objectives. | 1 |
| **INTRODUCTION** | | | |
| Rationale | 3 | Describe the rationale for the review in the context of what is already known. Explain why the review questions/objectives lend themselves to a scoping review approach. | 2 |
| Objectives | 4 | Provide an explicit statement of the questions and objectives being addressed with reference to their key elements (e.g., population or participants, concepts, and context) or other relevant key elements used to conceptualize the review questions and/or objectives. | 3 |
| **METHODS** | | | |
| Protocol and registration | 5 | Indicate whether a review protocol exists; state if and where it can be accessed (e.g., a Web address); and if available, provide registration information, including the registration number. | NA |
| Eligibility criteria | 6 | Specify characteristics of the sources of evidence used as eligibility criteria (e.g., years considered, language, and publication status), and provide a rationale. | 3-4 |
| Information sources* | 7 | Describe all information sources in the search (e.g., databases with dates of coverage and contact with authors to identify additional sources), as well as the date the most recent search was executed. | 3 |
| Search | 8 | Present the full electronic search strategy for at least 1 database, including any limits used, such that it could be repeated. | 3 |
| Selection of sources of evidence† | 9 | State the process for selecting sources of evidence (i.e., screening and eligibility) included in the scoping review. | 3,5 |
| Data charting process‡ | 10 | Describe the methods of charting data from the included sources of evidence (e.g., calibrated forms or forms that have been tested by the team before their use, and whether data charting was done independently or in duplicate) and any processes for obtaining and confirming data from investigators. | 3-5 |
| Data items | 11 | List and define all variables for which data were sought and any assumptions and simplifications made. | 3 |
| Critical appraisal of individual sources of evidence§ | 12 | If done, provide a rationale for conducting a critical appraisal of included sources of evidence; describe the methods used and how this information was used in any data synthesis (if appropriate). | NA |
| Synthesis of results | 13 | Describe the methods of handling and summarizing the data that were charted. | 3-4 |
| **RESULTS** | | | |
| Selection of sources of evidence | 14 | Give numbers of sources of evidence screened, assessed for eligibility, and included in the review, with reasons for exclusions at each stage, ideally using a flow diagram. | 4 |
| Characteristics of sources of evidence | 15 | For each source of evidence, present characteristics for which data were charted and provide the citations. | 4 |
| Critical appraisal within sources of evidence | 16 | If done, present data on critical appraisal of included sources of evidence (see item 12). | NA |
| Results of individual sources of evidence | 17 | For each included source of evidence, present the relevant data that were charted that relate to the review questions and objectives. | 4-10 |
| Synthesis of results | 18 | Summarize and/or present the charting results as they relate to the review questions and objectives. | 4-10 |
| **DISCUSSION** | | | |
| Summary of evidence | 19 | Summarize the main results (including an overview of concepts, themes, and types of evidence available), link to the review questions and objectives, and consider the relevance to key groups. | 5-11 |
| Limitations | 20 | Discuss the limitations of the scoping review process. | 12 |
| Conclusions | 21 | Provide a general interpretation of the results with respect to the review questions and objectives, as well as potential implications and/or next steps. | 12 |
| **FUNDING** | | | |
| Funding | 22 | Describe sources of funding for the included sources of evidence, as well as sources of funding for the scoping review. Describe the role of the funders of the scoping review. | 12 |

JBI = Joanna Briggs Institute; PRISMA-ScR = Preferred Reporting Items for Systematic reviews and Meta-Analyses extension for Scoping Reviews.

* Where *sources of evidence* (see second footnote) are compiled from, such as bibliographic databases, social media platforms, and Web sites.

† A more inclusive/heterogeneous term used to account for the different types of evidence or data sources (e.g., quantitative and/or qualitative research, expert opinion, and policy documents) that may be eligible in a scoping review as opposed to only studies. This is not to be confused with *information sources* (see first footnote).

‡ The frameworks by Arksey and O’Malley (6) and Levac and colleagues (7) and the JBI guidance (4, 5) refer to the process of data extraction in a scoping review as data charting*.*

§ The process of systematically examining research evidence to assess its validity, results, and relevance before using it to inform a decision. This term is used for items 12 and 19 instead of "risk of bias" (which is more applicable to systematic reviews of interventions) to include and acknowledge the various sources of evidence that may be used in a scoping review (e.g., quantitative and/or qualitative research, expert opinion, and policy document).
